# Primary nitrate responses mediated by calcium signalling and diverse protein phosphorylation

**DOI:** 10.1093/jxb/eraa047

**Published:** 2020-01-27

**Authors:** Kun-Hsiang Liu, Andrew Diener, Ziwei Lin, Cong Liu, Jen Sheen

**Affiliations:** 1 State Key Laboratory of Crop Stress Biology for Arid Areas and College of Life Sciences, Northwest Agriculture & Forestry University, Yangling, Shaanxi, China; 2 Department of Molecular Biology and Centre for Computational and Integrative Biology, Massachusetts General Hospital, and Department of Genetics, Harvard Medical School, Boston, MA, USA; 3 Nanjing Agricultural University, China

**Keywords:** Calcium signalling, nitrate signalling, primary nitrate response, protein kinase, protein phosphatase, transcription factor

## Abstract

Nitrate, the major source of inorganic nitrogen for plants, is a critical signal controlling nutrient transport and assimilation and adaptive growth responses throughout the plant. Understanding how plants perceive nitrate and how this perception is transduced into responses that optimize growth are important for the rational improvement of crop productivity and for mitigating pollution from the use of fertilizers. This review highlights recent findings that reveal key roles of cytosolic–nuclear calcium signalling and dynamic protein phosphorylation via diverse mechanisms in the primary nitrate response (PNR). Nitrate-triggered calcium signatures as well as the critical functions of subgroup III calcium-sensor protein kinases, a specific protein phosphatase 2C, and RNA polymerase II C-terminal domain phosphatase-like 3 are discussed. Moreover, genome-wide meta-analysis of nitrate-regulated genes encoding candidate protein kinases and phosphatases for modulating critical phosphorylation events in the PNR are elaborated. We also consider how phosphoproteomics approaches can contribute to the identification of putative regulatory protein kinases in the PNR. Exploring and integrating experimental strategies, new methodologies, and comprehensive datasets will further advance our understanding of the molecular and cellular mechanisms underlying the complex regulatory processes in the PNR.

## Teaser

Unique calcium signalling, specific calcium-sensor protein kinases, and diverse protein phosphorylation mechanisms modulate nutrient transport and assimilation, metabolism, hormone signalling and transcription in the primary nitrate responses in Arabidopsis .

## Introduction

Plant growth requires a source of nitrogen for the biosynthesis of amino acids, nucleic acids, and other nitrogen-containing biomolecules, and insufficient nitrogen reduces the productivity and quality of crops (Xu *et al.*, 2012; Bloom, 2015; Kiba and Krapp, 2016; Wang *et al.*, 2018; Fredes *et al.*, 2019). While nitrate and ammonium are major sources of inorganic nitrogen in soil (Wang *et al.*, 2018), most photosynthetic plants favour the assimilation of nitrate (Stitt, 1999; Crawford and Forde, 2002). In addition to being a key nutrient, nitrate acts as a signalling molecule and modulates nutrient uptake, assimilation, and metabolism. Nitrate signalling also controls morphological and physiological responses throughout the plant. Adaptation to fluctuations in available nitrate involves multifaceted responses in distinct developmental programmes controlling root system architecture, shoot morphology, seed germination, stomatal closure, and flowering time (Guo *et al.*, 2003; Dechorgnat *et al.*, 2011; Lin and Tsay, 2017; Liu *et al.*, 2017; Fredes *et al.*, 2019). For example, the root-system architecture of *Arabidopsis thaliana* is highly plastic in response to external nitrate levels. While nitrate starvation arrests root growth, a moderate level of nitrate in the environment promotes the elongation of lateral roots whose growth would be attenuated in high-nitrate conditions (Zhang and Forde, 2000; Linkohr *et al.*, 2002; Liu *et al.*, 2017).

Nitrate signalling orchestrates the adaptive responses of diverse biological processes through reprogramming of the transcriptome (Wang *et al.*, 2000, 2003, 2018; Scheible *et al.*, 2004; Canales *et al.*, 2014; Medici and Krouk, 2014; Vidal *et al.*, 2015; Liu *et al.*, 2017). The global transcriptional response to nitrate in Arabidopsis has been extensively investigated over the past few decades. These studies provide comprehensive information on the identity and role of selected transcription factors (TFs), including NIN-like proteins (NLPs), that can partially account for the changes in gene expression in the primary nitrate response (PNR) (Konishi and Yanagisawa, 2013; Marchive *et al.*, 2013; Vidal *et al.*, 2015; Liu *et al.*, 2017; Gaudinier *et al.*, 2018; Varala *et al.*, 2018; Brooks *et al.*, 2019). However, the primary signalling mechanisms that connect nitrate transporters/sensors to nitrate-mediated transcription and other biological processes remain mostly elusive.

Recent findings have provided new insights into how nitrate triggers dynamic changes in intracellular calcium signalling and protein phosphorylation to generate rapid control over nitrate uptake and the transcriptional PNR (Ho *et al.*, 2009; Hu *et al.*, 2009; Engelsberger and Schulze, 2012; Léran *et al.*, 2015; Riveras *et al.*, 2015; Menz *et al.*, 2016; Liu *et al.*, 2017). Although calcium signals with distinct amplitudes, locations (in the cytosol or other organelles), and durations are common features of plant responses to intrinsic or environmental cues (Dodd *et al.*, 2010), it has been challenging to demonstrate the precise calcium signatures triggered by nitrate due to technical limitations. In this review, we discuss the new evidence for nitrate-associated calcium signalling dynamics and regulatory mechanisms in the Arabidopsis PNR (Ho *et al.*, 2009; Hu *et al.*, 2009; Léran *et al.*, 2015; Riveras *et al.*, 2015; Liu *et al.*, 2017).

**Fig. 1.**
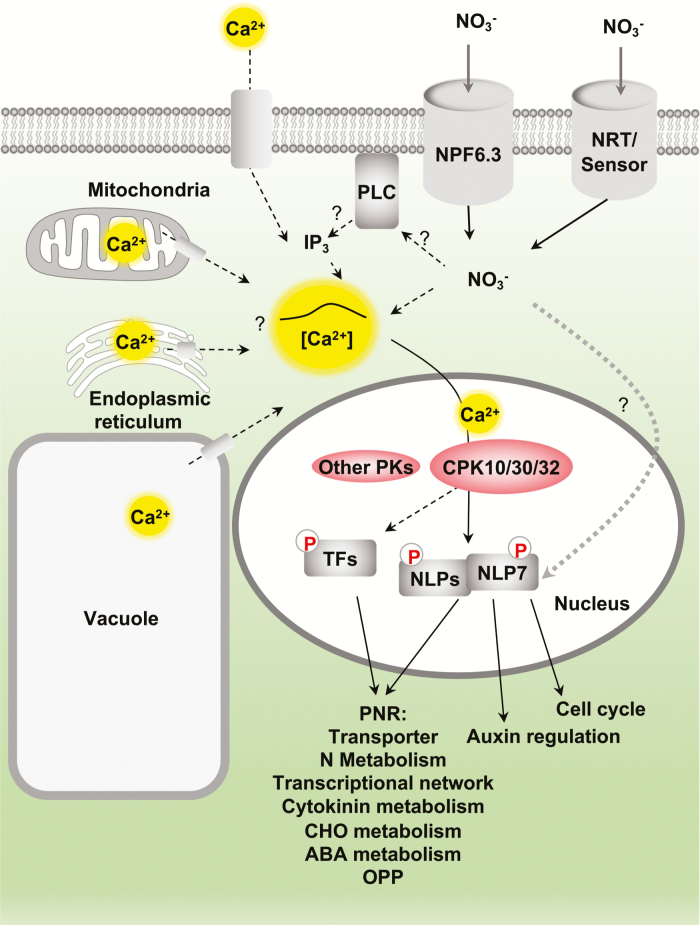
Calcium–CPK–NLP in nitrate signalling. Nitrate triggers a gradually rising calcium transient in the cytosol and nucleus. The involvement of phospholipase C (PLC)–inositol 1,4,5-trisphosphate (IP_3_) and the external or internal sources responsible for nitrate-triggered calcium increase are unclear. Increased calcium activates calcium-sensor protein kinases (CPKs), including CPK10, CPK30, and CPK32, which then phosphorylate NLP7 or other NLPs and transcription factors (TFs). This calcium–CPK–NLP signalling cooperates with the unknown nitrate-responsive signal (grey dotted arrow) to control gene expression in the primary nitrate response (PNR) as well as the NLP7-specific target genes mediating cell cycle and auxin regulation. Dashed arrows represent proposed connections. ABA, Abscisic acid; CHO, carbohydrate; NRT, Nitrate transporter; OPP, oxidative pentose phosphate pathway.

**Fig. 2.**
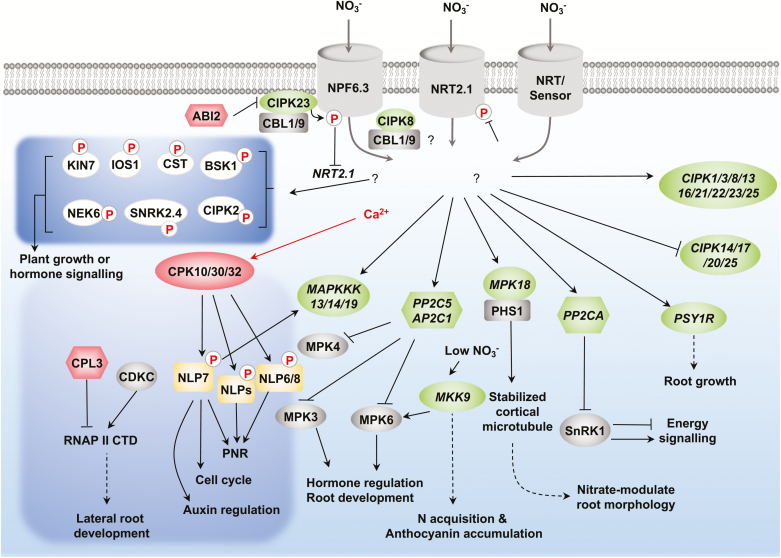
Diverse mechanisms modulate protein kinases (PKs) and protein phosphatases (PPs) in the primary nitrate response (PNR). At the transcriptional level (green), nitrate up- or down-regulates the expression of genes encoding CIPKs, MAPKKKs, PP2Cs, MKK9, MPK18, and PSY1R. Their downstream target proteins or partners are shown (grey). At the post-transcriptional level, nitrate activates CPK10/CPK30/CPK32, which then phosphorylate and activate NLP7, and possibly NLP6/8 and other NLPs (yellow). CPL3 might dephosphorylate the CTD domain of RNA polymerase II to negatively regulate its activity for genes involved in lateral root suppression. ABI2 interacts with and dephosphorylates the CIPK23–CBL1/CBL9 complex to regulate NPF6.3-mediated nitrate transport and signalling. The PNR induces phosphorylation of PKs (white ovals) and reduces phosphorylation of NRT2.1. The dashed arrows indicate proposed connections. ABI2; ABA-INSENSITIVE 2; AP2C1, ARABIDOPSIS SER/THR PHOSPHATASE TYPE 2C 1; BSK1, BR-SIGNALLING KINASE 1; CBL, calcineurin B-like protein; CDKC, cyclin-dependent kinase C; CIPK, calcineurin B-like protein-interacting protein kinase; CPK, calcium-sensor protein kinase; CPL3, RNA POLYMERASE II C-TERMINAL DOMAIN PHOSPHATASE-LIKE 3; CST, CAST AWAY; CTD, C-terminal domain; IOS1, IMPAIRED OOMYCETE SUSCEPTIBILITY 1; KIN7, KINASE 7; MAPK, mitogen-activated PK; MAPKK, MAPK kinase; MAPKKK, MAPKK kinase; NEK6, NEVER IN MITOSIS GENE A-RELATED KINASE 6; NLP, NIN-Like Protein; NPF, nitrate transporter 1/ peptide transporter family; NRT, nitrate transporter; PHS1, PROPYZAMIDE HYPERSENSITIVE 1; PP2C, protein phosphatase C; PSY1R, PLANT PEPTIDE CONTAINING SULFATED RECEPTOR; RNA II, RNA polymerase II; SNRK2.4, SUCROSE NONFERMENTING 1-RELATED KINASE 2.4.

In addition to transcriptional regulation, nitrate signalling involves post-translational modifications such as protein phosphorylation, protein ubiquitination, and chromatin modification (Widiez *et al.*, 2011; Sato *et al.*, 2009; Liu *et al.*, 2017; Alvarez *et al.*, 2019; Hu *et al.*, 2019; Poza-Carrion and Paz-Ares, 2019). In particular, protein phosphorylation can lead to rapid, versatile, and reversible modifications that directly regulate the localization, stability, interaction, function, and enzymatic activity of target proteins (Yip Delormel and Boudsocq, 2019). Here, we highlight the significance of nitrate-triggered calcium signalling, nitrate-activated calcium-sensor protein kinases (CPKs), and the nitrate–CPK–NLP regulatory network (Fig. 1) (Ho *et al.*, 2009; Hu *et al.*, 2009; Léran *et al.*, 2015; Riveras *et al.*, 2015; Liu *et al.*, 2017). In addition, we review the recently defined roles for ABA-INSENSITIVE 2 (ABI2) (Léran *et al.*, 2015) and C-TERMINAL DOMAIN (CTD) PHOSHATASE-LIKE 3 (CPL3) (Liu *et al.*, 2012), as well as the potential functions of nitrate-responsive genes encoding candidate protein kinases (PKs) and protein phosphatases (PPs) in the Arabidopsis PNR. Finally, we consider the use of phosphoproteomics approaches to identify nitrate-modulated protein phosphorylation targeting putative PKs (Fig. 2). These emerging discoveries clearly indicate the enormous complexity of the signalling network involving multifaceted calcium and protein phosphorylation regulations in the PNR.

## Nitrate-triggered calcium signalling in the PNR

The involvement of calcium and protein phosphorylation in nitrate signalling was first suggested over 20 years ago (Sakakibara *et al.*, 1997). Experiments in which detached maize and barley leaves were treated with chemical inhibitors of PKs or PPs, calcium chelators, or calcium channel blockers have suggested that calcium, PKs, and PPs are critical to nitrate-responsive gene transcription (Sakakibara *et al.*, 1997; Sueyoshi *et al.*, 1999). Using Arabidopsis seedlings, it has recently been shown that pre-treatment with gadolinium ions and lanthanide ions (which act as calcium channel blockers) or W7, an inhibitor of intracellular calmodulin and CPK, significantly reduces the nitrate-stimulated expression of marker genes. However, the findings also suggest the existence of calcium-dependent and calcium-independent pathways in nitrate-responsive gene regulation (Riveras *et al.*, 2015; Liu *et al.*, 2017). Despite the biochemical and molecular evidence for the involvement of calcium in nitrate-regulated gene expression, there has been no report on cellular observation of a defined calcium signature triggered by nitrate until very recent studies using various calcium biosensors in plants (Riveras *et al.*, 2015; Liu *et al.*, 2017).

## Nitrate-associated calcium signalling dynamics

The nitrate-triggered changes in intracellular calcium were first reported in studies using plants expressing the aequorin reporter gene, which showed that nitrate stimulates a rapid calcium spike within 10 s in excised roots. This rapid cytoplasmic calcium signal is abolished in the roots of NITRATE TRANSPORTER 1/PEPTIDE TRANSPORTER FAMILY 6.3 (NPF6.3)/CHLORATE-RESISTANT 1 (CHL1)/NITRATE TRANSPORTER 1.1 (NRT1.1) (hereafter NPF6.3) mutants *chl1-5* and *chl1-9*, indicating that the nitrate transceptor is responsible for this very transient calcium spike (Riveras *et al.*, 2015). However, in intact Arabidopsis seedlings, the nitrate-stimulated calcium dynamics appear dissimilar when using the same aequorin reporter (Liu *et al.*, 2017). In the whole seedling, nitrate and potassium chloride (as a control for signal specificity) do not trigger detectable changes in intracellular calcium before 10 s. Nitrate specifically stimulates a subtle and gradual increase in calcium, peaking at around 100 s (Liu *et al.*, 2017).

Because the nitrate-activated calcium signal in aequorin-expressing plants is much weaker than that stimulated by the bacterial flagellin peptide flg22 (Liu *et al.*, 2017), a new generation of genetically encoded calcium biosensors, such as the ultrasensitive GCaMP6s (Chen *et al.*, 2013), are needed to more clearly visualize the calcium dynamics triggered by nitrate. Furthermore, the use of GCaMP6s enables the quantification of calcium dynamics at subcellular resolution in single cells, which cannot be achieved with the aequorin reporter. In isolated leaf cells co-expressing GCaMP6s and a nuclear mCherry marker, time-lapse recordings in single cells reveal that nitrate specifically stimulates a unique calcium signature in the nucleus and cytosol. Nitrate triggers a similarly gradual increase in calcium signals, peaking at 2–3 min, in the mesophyll cells of intact transgenic cotyledons. In the root cap and root stele of intact GCaMP6s-expressing plants, the nitrate-induced calcium signal peaks at around 50–90 s (Liu *et al.*, 2017). The calcium-binding dissociation constant (*K*_d_) for GCaMP6s is 144 nM, just above the resting calcium level of ~100 nM in plant cells, whereas the calcium-binding *K*_d_ for aequorin is around 7.2–13 μM (Chen *et al.*, 2013; Costa *et al.*, 2018). Because the increase in intracellular calcium triggered by nitrate is relatively modest, the use of a calcium biosensor of appropriate sensitivity is critical for detecting a physiologically relevant link to calcium (Liu *et al.*, 2017).

Dynamic fluorescent imaging using GCaMP6s reveals that the relatively weak calcium signals induced by nitrate are clearly distinct from the strong but very transient (lasting less than 60 s) cytoplasmic calcium spike triggered by cold or osmotic stress (Knight *et al.*, 1996; Yuan *et al.*, 2014), or the strong and sustained calcium flood induced by microbial signals or effectors, which persists for 20–120 min (Grant *et al.*, 2000; Ranf *et al.*, 2015; Liu *et al.*, 2017). Visualization of more modest calcium signals is now possible with high reproducibility using GCaMP6 and GCaMP7, which surpass the sensitivity of other protein-based and commonly used synthetic calcium indicators (Chen *et al.*, 2013; Dana *et al.*, 2019). Such calcium biosensors have lower limits of detection and higher resolution than previously used biosensors, and enable the monitoring of physiological and dynamic calcium signalling triggered by diverse nutrients, peptides, and hormones at subcellular resolution in plants and animals. Future research will determine the precise sources and subcellular localization of nitrate-stimulated calcium signals using sensitive calcium biosensors targeted to specific organelles or subcellular compartments, which should also facilitate the identification of physiologically relevant calcium channels as well as additional nitrate and calcium sensors in the PNR (Fig. 1).

## Mechanisms of calcium regulation

Inositol 1,4,5-trisphosphate (IP_3_) plays a critical role in triggering calcium signals by regulating calcium-selective intracellular channels in eukaryotic cells (Berridge, 2016). Although IP_3_ receptors have not been identified in plants, a positive correlation between increasing IP_3_ levels in the cell and elevated calcium signals has been suggested (Singh *et al.*, 2015). Because phospholipase C (PLC) releases IP_3_ and diacylglycerol from phosphatidylinositol 4,5-bisphosphate [PI(4,5)P_2_] in plants (Kanehara *et al.*, 2015), the effect of the PLC inhibitor U73122 on nitrate signalling has been examined. As U73122 inhibits nitrate-induced marker gene expression and IP_3_ accumulates at 10 s of nitrate induction, it has been suggested that PLC–IP_3_ plays a critical role in nitrate signalling. However, the IP_3_ accumulation detected at 10 s is not followed by a further increase of calcium signalling in excised Arabidopsis roots (Riveras *et al.*, 2015). Further biochemical, cellular, and genetic studies are needed to connect these promising findings of a link between nitrate regulation of PLC–IP_3_ and calcium signalling.

Interestingly, nitrate induces the expression of a gene encoding myo-inositol polyphosphate 5-phosphatase2 (5-PTase2), which has the ability to remove 5′ phosphate from IP_3_ and inositol 1,3,4,5-tetrakisphosphate (Ins(1,3,4,5)P_4_) *in vitro* (Berdy *et al.*, 2001; Canales *et al.*, 2014). This suggests that nitrate may induce 5-PTase2 and negatively regulate IP_3_ accumulation in the cell. Prior studies have shown that IP_3_ accumulates in both *5ptase1* and *5ptase2* mutants (Gunesekera *et al.*, 2007), and plants constitutively expressing the human type I 5-PTase have a reduced basal IP_3_ level (Perera *et al.*, 2006). Nitrate-induced *5-PTase2* gene expression may contribute to the control of IP_3_ homeostasis by a negative feedback mechanism. Elucidating the connections between nitrate, the nitrate transceptor, PLC, IP_3_, and calcium signalling may open up a new direction for studying the PNR (Fig. 1).

## The nitrate–CPK–NLP signalling network

As both calcium and protein phosphorylation were implicated in early nitrate signalling, an in-gel kinase assay was conducted to determine whether putative calcium-regulated PKs could be activated by nitrate (Liu *et al.*, 2017). The estimated molecular weight of nitrate-activated PKs was approximately 60 kDa, which is similar to the predicted molecular size of most CPKs but larger than the calcineurin B-like protein (CBL)-interacting PKs (CIPKs) previously reported to regulate nitrate transport and signalling in Arabidopsis (Ho *et al.*, 2009; Hu *et al.*, 2009; Léran *et al.*, 2015; Liu *et al.*, 2017). A targeted functional screen for Arabidopsis CPKs that enhance the expression of a nitrate-responsive reporter gene in the presence of very low nitrate to sensitize the response led to the identification of the functionally redundant subgroup III CPK10/30/32 as new regulators in the PNR. Because single *cpk* mutants lack overt growth phenotypes and the *cpk10,30* double mutant exhibits embryonic lethality, a chemical genetic approach was developed to generate a so-called ‘inducible *cpk10,30,32*’ (*icpk*) triple mutant (Liu *et al.*, 2017). In the *icpk* mutant, a transgene was introduced to express the CPK10(M141G) variant protein that can be reversibly inhibited by a selective and potent PK inhibitor analogue, 3MB-PP1-IsoP (Liu *et al.*, 2017). Thus, chemical genetics circumvents lethality and redundancy, and offers a new strategy to further elucidate the dynamic and physiological functions of higher-order *cpk* mutants.

Integrated transcriptomic and phenotypic analyses of wild-type and *icpk* plants indicate that CPK10/30/32 control nitrate-specific gene expression and growth. CPK10/30/32 act as functionally redundant master regulators, modulating a broad spectrum of genes involved in diverse cellular and metabolic pathways immediately activated by nitrate. For example, target genes of CPK10/30/32 are enriched for functions in: nitrate/ammonia transport and assimilation; two routes of glucose-6-phosphate metabolism via the oxidative pentose phosphate pathway and glycolysis; amino acid transport and metabolism; cell wall remodelling; other transporters; carbon/nitrogen metabolism; cytokinin, auxin, and abscisic acid (ABA) metabolism and signalling; protein degradation; stress; signalling; and transcription. Notably, the expression of genes encoding functionally important TFs such as basic region/leucine zipper motif (bZIP), MYB, basic helix-loop-helix (bHLH), LATERAL ORGAN BOUNDARIES (LBD), SQUAMOSA PROMOTER BINDING PROTEIN-LIKE (SPL), WRKY, HYPERSENSITIVITY TO LOW PI-ELICITED PRIMARY ROOT SHORTENING 1 (HRS1), and TGACG MOTIF-BINDING FACTOR (TGA) is induced by nitrate-activated CPK10/30/32 to support potential amplification of the downstream nitrate transcriptional network (Liu *et al.*, 2017; Vidal *et al.*, 2015; Wang *et al.*, 2018). Moreover, nitrate–CPK signalling activates an Arabidopsis gene encoding the cytochrome P450 enzyme CYP735A2, which directly enhances *trans*-zeatin synthesis to promote shoot development and provides an interconnection between local and systemic nitrate signalling via the action of a mobile growth hormone (Fig. 1) (Kiba *et al.*, 2013; Liu *et al.*, 2017).

Importantly, CPK10/30/32 can directly phosphorylate Ser-205 of NLP7 and activate this master TF in the PNR. It has been demonstrated that the phosphorylation of NLP7 by CPK10/30/32 is calcium dependent and leads to nuclear retention and activation of NLP7 (Marchive *et al.*, 2013; Liu *et al.*, 2017). A previous study suggested that subgroup III CPKs are relatively insensitive to calcium in an *in vitro* assay for kinase activity. However, the kinase activity assay was conducted using a washing buffer containing ~600 nM calcium, which may be sufficient to activate subgroup III CPKs (Boudsocq *et al.*, 2012). Consistently, the calcium-binding *K*_d_ of GCaMP6s required for the clear detection of the specific calcium increase stimulated by nitrate appears to match the calcium sensitivity of nitrate-activated CPKs (Chen *et al.*, 2013; Costa *et al.*, 2018).

Transient expression of ectopic NLP7, but not NLP7(S205A), in mesophyll protoplasts is sufficient to activate a wide range of putative NLP7 target genes beyond the well-known nitrate-responsive marker genes (Liu *et al.*, 2017). These novel CPK–NLP7 target genes include S phase genes involved in cell cycle initiation as well as genes involved in auxin synthesis, transport, and signalling. These new CPK–NLP7–auxin pathways can be mechanistically separated from the NPF6.3-dependent auxin transport function (Krouk *et al.*, 2010*a*) or the nitrate-stimulated expression of *AFB3*, which encodes an auxin receptor (Vidal *et al.*, 2010; O’Brien *et al.*, 2016). Notably, auxin biosynthesis mediated by TRYPTOPHAN AMINOTRANSFERASE RELATED 2 (TAR2) in root meristem cells and auxin signalling mediated by a RECEPTOR-LIKE KINASE (RLK) TRANSMEMBRANE KINASE 1 (TMK1) Transmembrane kinase 1 are likely crucial for the promotion of lateral root development by nitrate (Dai *et al.*, 2013; Ma *et al.*, 2014). These unique NLP7 target genes may partially explain the specific growth defects found in *nlp7* but not other *nlp* single mutant plants, which can be fully rescued by NLP7 but not NLP(S205A) (Liu *et al.*, 2017). Further studies of these novel NLP7 target genes in response to nitrate–CPK signalling may expand our understanding of nitrate-modulated development in specific cell types of different plant organs.

CPKs are the emerging master regulator targeting a broad range of cellular substrates in diverse signalling pathways (Curran *et al.*, 2011; Boudsocq and Sheen, 2013; Simeunovic *et al.*, 2016; Yip Delormel and Boudsocq, 2019). As the CPK-phosphorylated Ser defined in NLP7 is conserved in Arabidopsis NLP1-9 and in NLP orthologs of different plant species, CPK10/30/32 may regulate other NLPs for common or distinct target genes in diverse cell types for different developmental processes in the PNR (Konishi and Yanagisawa, 2013; Marchive *et al.*, 2013; Yan *et al.*, 2016; Liu *et al.*, 2017). It is possible that more TFs are substrates of CPKs to mediate gene regulation in the PNR, or CPKs may also phosphorylate transporters, channels, and enzymes to exert broader controls in the response to nitrate. The manipulation of specific CPKs may facilitate targeted plant modification and improvement for multiple traits beneficial in terms of nutrient utilization, stress tolerance, and pathogen resistance (Boudsocq and Sheen, 2013; Simeunovic *et al.*, 2016; Liu *et al.*, 2017; Yip Delormel and Boudsocq, 2019). Further exploration of the nitrate–CPK–NLP signalling network will provide new insight into the nutrient–growth signalling pathways and the importance of post-translational modifications in nitrate signalling (Fig. 1).

## ABI2–CBL1/9–CIPK23 signalling in NPF6.3-dependent nitrate responses

NPF6.3 is a dual-affinity nitrate transporter and a nitrate sensor (Liu and Tsay, 2003; Ho *et al.*, 2009). The high-affinity mode of NPF6.3 function in nitrate transport is switched on by CIPK23 phosphorylation of Thr101 in the presence of low external nitrate, which also inhibits the PNR mediated by NPF6.3 based on *Nitrate transporter 2.1* (*NRT2.1*) activation (Ho *et al.*, 2009; Liu *et al.*, 2017). The putative calcium sensors CBL1 and CBL9 interact with CIPK23 to activate kinase activity (Ho *et al.*, 2009; Léran *et al.*, 2015; Bender *et al.*, 2018). To identify PPs that counteract the inhibitory effect of CBL1–CIPK23 on NPF6.3-depedent nitrate transport activity, co-expression of PP2Cs belonging to clade A was examined in *Xenopus* oocytes. ABI2 was identified as a specific PP2C that can antagonize CBL1–CIPK23 inhibition of NPF6.3-mediated nitrate uptake in the oocyte assay, which was supported by the characterization of nitrate influx in different PP2C mutant plants (Léran *et al.*, 2015).

ABI2 regulates nitrate-mediated *NRT2-1* expression and root growth by dephosphorylating CBL1 and CIPK23 and enhancing NPF6.3 activity in nitrate signalling (Léran *et al.*, 2015). It will be interesting to determine whether different concentrations of nitrate trigger differential calcium signals to modulate CPK10/30/32 as positive regulators (Fig. 1) and CBL1/9–CIPK23 as negative regulators (Fig. 2) in the PNR. Besides inhibiting NPF6.3, CBL1/9–CIPK23 has been shown to activate the potassium channel AKT1, although a role in calcium signalling remains unclear (Xu *et al.*, 2006; Bender *et al.*, 2018). It has been proposed that drought and osmotic stress induce ABA accumulation and then inactivate ABI2, thereby enhancing the uptake of potassium ions as cellular osmolytes and reducing the uptake of nitrate to shut down energy-consuming processes of nitrate reduction (Léran *et al.*, 2015). The ABI2–CBL1/9–CIPK23 link shows how stresses affect nutrient uptake and utilization at the level of post-translational regulation. Whether subcellular compartmentalization or substrate specificity distinguishes ABI2 from other related clade A PP2Cs in exerting its unique regulation of different ion channels/transporters deserves further research.

## CPL3 as a regulator in nitrate responses

A forward genetic screen based on two consecutive assays, the first of nitrate-responsive reporter gene expression and the second of plant growth phenotypes, has led to the isolation of *nis* (*nitrate insensitive*) and *ncr* (*nitrate constitutive response*) mutants. The *ncr1* mutant exhibits elevated expression of the nitrite reductase gene (*NIR*) in the absence of nitrate and lacks the normal inhibitory effect of high nitrate on lateral root elongation (Liu *et al.*, 2012). *NCR1* encodes CPL3, which has functions in multiple regulatory pathways, for example, as a negative regulator of immune gene expression, stress-responsive gene transcription, or plant development (Bang *et al.*, 2006; Li *et al.*, 2014).

The RNA polymerase II complex is composed of multiple evolutionarily conserved subunits and is responsible for the transcription of protein-coding genes in all eukaryotes. The CTD of the largest subunit consists of conserved heptapeptide repeats with the consensus sequence Y_1_S_2_P_3_T_4_S_5_P_6_S_7_ (Buratowski, 2009). It has been demonstrated that CPL3 dephosphorylates Ser-2 to suppress CTD phosphorylation by mitogen-activated PK (MAPK) and cyclin-dependent kinase C (CDKC). Based on the transcriptome analysis of plant immune responses in wild type and *cpl3*, CPL3 does not control general gene transcription but participates in the regulation of specific flg22-responsive gene expression (Li *et al.*, 2014). The molecular mechanism of high-nitrate inhibition of lateral root elongation is unclear. It is possible that CPL3 may mediate high-nitrate suppression of genes involved in regulating lateral root elongation. Whether NCR1 specifically or broadly regulates gene expression in the PNR or affects the expression of genes involved in modulating lateral root elongation through the modulation of RNA polymerase II activity will require further molecular and genomic dissection.

## Nitrate-regulated genes encoding candidate PKs and PPs in the PNR

Over the past two decades, independent studies have investigated nitrate-regulated global transcription using various microarray platforms in Arabidopsis with different nitrate concentrations, at different response time points, in different plant organs, and at different developmental stages using various growth conditions (Wang *et al.*, 2000, 2003, 2004, 2007; Gutiérrez *et al*., 2007; Gifford *et al.*, 2008; Hu *et al.*, 2009; Krouk *et al.*, 2010*b*; Patterson *et al.*, 2010; Ruffel *et al.*, 2011; Canales *et al.*, 2014; Vidal *et al.*, 2015). Recent studies using RNA sequencing (RNA-seq) capture the expression of more genes and better resolve differential expression in the PNR (Li *et al.*, 2017; Liu *et al.*, 2017). These comprehensive transcriptome datasets are rich information resources for constructing integrated nitrate signalling and gene regulatory networks based on TF–target gene relationships and for identifying candidate signalling components (Canales *et al.*, 2014; Vidal *et al.*, 2015).

To evaluate the hypothesis that additional nitrate-responsive genes encoding PKs and PPs are involved in nitrate signalling, we surveyed the differentially expressed genes identified in a published meta-analysis of transcriptomic data derived from Affymetrix microarrays and two datasets from more recent RNA-seq analyses (Canales *et al.*, 2014; Li *et al.*, 2017; Liu *et al.*, 2017). We ranked nitrate-induced or nitrate-repressed genes encoding PKs or PPs by the number of experiments in which they were detected as being nitrate responsive (Table 1). Interestingly, some of the most robustly enriched nitrate-regulated genes encode different CIPKs, putative MAPK cascade components, RLKs, and PP2Cs (Table 1) (Fig. 2). Although there is limited information on their physiological roles in nitrate signalling, we will discuss potentially relevant functions of these four major groups of PKs and PPs encoded by nitrate-responsive genes.

**Table 1.** Genome-wide meta-analysis of nitrate-responsive genes encoding protein kinases and protein phosphatases

Gene ID	Description	Total ^*a*^	Induced	Repressed
**AT2G26980**	**CBL-interacting protein kinase 3 (*CIPK3*)**	**20**	**20**	**0**
**AT3G16560**	**Protein phosphatase 2C family protein**	**16**	**16**	**0**
**AT4G32950**	**Protein phosphatase 2C family protein**	**15**	**15**	**0**
**AT4G38470**	**ACT-like protein tyrosine kinase family protein (*STY46*)**	**14**	**14**	**0**
**AT2G30040**	**Mitogen-activated protein kinase kinase kinase 14 (*MAPKKK14*)**	**14**	**14**	**0**
**AT1G49160**	**Protein kinase superfamily protein (*WNK7*)**	**14**	**14**	**0**
**AT3G17510**	**CBL-interacting protein kinase 1 (*CIPK1*)**	**12**	**12**	**0**
**AT5G26010**	**Protein phosphatase 2C family protein**	**11**	**2**	**9**
**AT5G54130**	**Calcium-binding endonuclease/exonuclease/phosphatase family**	**10**	**10**	**0**
**AT2G17820**	**Histidine kinase 1 (*HK1*)**	**9**	**9**	**0**
**AT1G07150**	**Mitogen-activated protein kinase kinase kinase 13 (*MAPKKK13*)**	**8**	**8**	**0**
**AT2G17700**	**ACT-like protein tyrosine kinase family protein (*STY8*)**	**7**	**0**	**7**
**AT1G30270**	**CBL-interacting protein kinase 23 (*CIPK23*)**	**6**	**6**	**0**
**AT2G23030**	**SNF1-related protein kinase 2.9 (*SNRK2.9*)**	**6**	**6**	**0**
**AT1G72300**	**Leucine-rich receptor-like protein kinase family protein (*PSY1R*)**	**5**	**5**	**0**
**AT3G11410**	**Protein phosphatase 2CA (*PP2CA*)**	**5**	**5**	**0**
**AT1G73500**	**MAP kinase kinase 9 (*MKK9*)**	**5**	**0**	**5**
**AT4G29990**	**Leucine-rich repeat transmembrane protein kinase protein**	**4**	**4**	**0**
**AT1G53510**	**Mitogen-activated protein kinase 18 (*MPK18*)**	**4**	**4**	**0**
**AT5G67080**	**Mitogen-activated protein kinase kinase kinase 19 (*MAPKKK19*)**	**4**	**4**	**0**
**AT3G22750**	**Protein kinase superfamily protein**	**4**	**4**	**0**
**AT2G30020**	**Protein phosphatase 2C family protein (*AP2C1*)**	**4**	**4**	**0**
**AT5G38240**	**Protein kinase family protein**	**4**	**2**	**2**
**AT1G33260**	**Protein kinase superfamily protein**	**4**	**0**	**4**
**AT2G34180**	**CBL-interacting protein kinase 13 (*CIPK13*)**	**3**	**3**	**0**
**AT2G25090**	**CBL-interacting protein kinase 16 (*CIPK16*)**	**3**	**3**	**0**
**AT4G21410**	**Cysteine-rich RLK (RECEPTOR-like protein kinase) 29 (*CRK29*)**	**3**	**3**	**0**
**AT4G11460**	**Cysteine-rich RLK (RECEPTOR-like protein kinase) 30 (*CRK30*)**	**3**	**3**	**0**
**AT3G58760**	**Integrin-linked protein kinase family (*ILK4*)**	**3**	**3**	**0**
**AT5G01820**	**CBL-interacting protein kinase 16 (*CIPK14*)**	**3**	**0**	**3**
AT1G76040	Calcium-dependent protein kinase 29 (*CPK29*)	2	2	0
AT2G38490	CBL-interacting protein kinase 22 (*CIPK22*)	2	2	0
AT1G49100	Leucine-rich repeat protein kinase family protein	2	2	0
AT1G51810	Leucine-rich repeat protein kinase family protein	2	2	0
AT5G62710	Leucine-rich repeat protein kinase family protein	2	2	0
AT2G28250	Protein kinase superfamily protein (*NCRK*)	2	2	0
AT3G07070	Protein kinase superfamily protein	2	2	0
AT3G28690	Protein kinase superfamily protein	2	2	0
AT1G63580	Receptor-like protein kinase-related family protein	2	2	0
AT1G05000	Phosphotyrosine protein phosphatases superfamily protein (*PFA-DSP1*)	2	2	0
AT1G16220	Protein phosphatase 2C family protein	2	2	0
AT3G16800	Protein phosphatase 2C family protein (*EGR3*)	2	2	0
AT5G25110	CBL-interacting protein kinase 25 (*CIPK25*)	2	1	1
AT5G59650	Leucine-rich repeat protein kinase family protein	2	1	1
AT1G07880	Protein kinase superfamily protein (*ATMPK13*)	2	1	1
AT3G59350	Protein kinase superfamily protein	2	1	1
AT3G14370	Protein kinase superfamily protein (*WAG2*)	2	1	1
AT2G40180	Phosphatase 2C5 (*PP2C5*)	2	1	1
AT1G51805	Leucine-rich repeat protein kinase family protein (*SIF3*)	2	0	2
AT2G23300	Leucine-rich repeat protein kinase family protein	2	0	2
AT4G33950	Protein kinase superfamily protein (*OST1*)	2	0	2
AT1G66930	Protein kinase superfamily protein (*LRK10L2*)	2	0	2
AT5G66210	Calcium-dependent protein kinase 28 (*CPK28*)	1	1	0
AT5G57630	CBL-interacting protein kinase 21 (*CIPK21*)	1	1	0
AT4G24400	CBL-interacting protein kinase 8 (*CIPK8*)	1	1	0
AT2G29220	Concanavalin A-like lectin protein kinase family protein (*LECRK-III.1*)	1	1	0
AT4G23230	Cysteine-rich RLK (RECEPTOR-like protein kinase) 15 (*CKR15*)	1	1	0
AT4G21400	Cysteine-rich RLK (RECEPTOR-like protein kinase) 28 (*CRK28*)	1	1	0
AT4G04490	Cysteine-rich RLK (RECEPTOR-like protein kinase) 36 (*CRK36*)	1	1	0
AT4G04540	Cysteine-rich RLK (RECEPTOR-like protein kinase) 39 (*CRK39*)	1	1	0
AT4G23140	Cysteine-rich RLK (RECEPTOR-like protein kinase) 6 (*CRK6*)	1	1	0
AT2G19190	FLG22-induced receptor-like kinase 1 (*SIRK*)	1	1	0
AT1G75820	Leucine-rich receptor-like protein kinase family protein (*CLV1*)	1	1	0
AT2G29000	Leucine-rich repeat protein kinase family protein	1	1	0
AT3G46370	Leucine-rich repeat protein kinase family protein	1	1	0
AT5G49780	Leucine-rich repeat protein kinase family protein	1	1	0
AT2G19210	Leucine-rich repeat transmembrane protein kinase protein	1	1	0
AT2G01450	MAP kinase 17 (*MPK17*)	1	1	0
AT5G54380	Protein kinase family protein (*THE1*)	1	1	0
AT3G63260	Protein kinase superfamily protein (*ATMRK1*)	1	1	0
AT1G68690	Protein kinase superfamily protein (*PERK9*)	1	1	0
AT1G66460	Protein kinase superfamily protein	1	1	0
AT5G41680	Protein kinase superfamily protein	1	1	0
AT4G11890	Protein kinase superfamily protein (*ARCK1*)	1	1	0
AT1G63550	Receptor-like protein kinase-related family protein	1	1	0
AT4G40010	SNF1-related protein kinase 2.7 (*SNRK2.7*)	1	1	0
AT5G27930	Protein phosphatase 2C family protein (*EGR2*)	1	1	0
AT1G07160	Protein phosphatase 2C family protein	1	1	0
AT2G33700	Protein phosphatase 2C family protein	1	1	0
AT1G48260	CBL-interacting protein kinase 17 (*CIPK17*)	1	0	1
AT5G45820	CBL-interacting protein kinase 20 (*CIPK20*)	1	0	1
AT3G45330	Concanavalin A-like lectin protein kinase family protein (*LECRK-I.1*)	1	0	1
AT5G01550	Lectin receptor kinase a4.1 (*LECRK-VI.3*)	1	0	1
AT5G01560	Lectin receptor kinase a4.3 (*LECRKA4.3*)	1	0	1
AT1G51890	Leucine-rich repeat protein kinase family protein	1	0	1
AT5G49770	Leucine-rich repeat protein kinase family protein	1	0	1
AT1G05700	Leucine-rich repeat transmembrane protein kinase protein	1	0	1
AT1G26150	Proline-rich extensin-like receptor kinase 10 (*PERK10*)	1	0	1
AT1G61590	Protein kinase superfamily protein	1	0	1
AT3G09010	Protein kinase superfamily protein	1	0	1
AT4G09760	Protein kinase superfamily protein (*CEK3*)	1	0	1
AT3G45920	Protein kinase superfamily protein	1	0	1
AT3G46270	Receptor protein kinase-related	1	0	1
AT5G10520	ROP binding protein kinases 1 (*RBK1*)	1	0	1
AT3G23150	Signal transduction histidine kinase, hybrid-type, ethylene sensor (*ETR2*)	1	0	1
AT1G61360	S-locus lectin protein kinase family protein	1	0	1
AT1G61500	S-locus lectin protein kinase family protein	1	0	1
AT4G27300	S-locus lectin protein kinase family protein	1	0	1
AT1G21230	Wall associated kinase 5 (*WAK5*)	1	0	1
AT2G32960	Phosphotyrosine protein phosphatases superfamily protein (*PFA-DSP2*)	1	0	1

Differential gene expression (Canales *et al.*, 2014; Li *et al.*, 2017; Liu *et al.*, 2017). In our analysis, we applied a ≥2-fold cut-off.

^*a*^ Total number of experiments in which expression of a gene is either induced or repressed by nitrate. Genes that are induced or repressed by nitrate in three or more experiments are in bold text.

### Distinct signalling by different CIPKs

The CBL–CIPK complexes play essential roles in regulating the homeostasis of intracellular potassium, sodium, and magnesium in stressed and unstressed plants (Zhu, 2003; Luan, 2009; Mao *et al.*, 2016; Bender *et al.*, 2018). Both *CIPK23* and *CIPK8* genes are activated by nitrate, although two CBL–CIPK modules, CBL1/9–CIPK23 and CBL1/9–CIPK8, have been shown to play differential roles in nitrate signalling. CBL1/9–CIPK8 positively regulates the low-affinity phase [with a Michaelis constant (*K*_m_) of ~0.9 mM nitrate] of the PNR, whereas CBL1/9–CIPK23 can response to low-nitrate conditions and phosphorylate Thr101 of NPF6.3, which negatively regulates the high-affinity phase (with a *K*_m_ of ~30 μM nitrate) of nitrate responses (Ho *et al.*, 2009; Hu *et al.*, 2009). Whether different nitrate levels could trigger differential calcium dynamics, which are decoded by CIPK8 and CIPK23 associated with the NPF6.3 transceptor, to mediate their distinct regulatory roles in the PNR remains to be resolved (Fig. 2).

In the Arabidopsis genome, 26 *CIPK* and 10 *CBL* genes encode members of the CIPK and CBL protein families, respectively (Kolukisaoglu *et al.*, 2004; Mao *et al.*, 2016). Depending on the experimental conditions, nitrate may induce the expression of *CIPK1*, *3*, *8*, *13*, *14*, *16*, *21*, *22*, *23*, and *25* or repress the expression of *CIPK17*, *20*, and *25*. *CIPK1* and *CIPK3* have been consistently induced by nitrate in numerous experiments (Table 1), but the characterization of *cipk1* and *cipk3* mutants has not yielded any clue as to their physiological roles in the PNR (Hu *et al.*, 2009). As *cipk3* is hypersensitive to ABA during germination (Pandey *et al.*, 2008), CBL9–CIPK3 may mediate the nitrate promotion of germination that is antagonized by ABA. Further analysis using the *cipk1,3* double mutant or even higher-order *cipk* mutants may be necessary to elucidate the functions of CIPK1, CIPK3, or other CIPKs in nitrate signalling (Fig. 2) (Kolukisaoglu *et al.*, 2004).

### Potential functions of MAPK cascades

The evolutionarily conserved MAPK cascades consist of upstream MAPK kinase kinases (MAPKKKs) that phosphorylate MAPK kinases (MAPKKs), which in turn phosphorylate MAPKs. Hundreds of protein substrates can be potentially phosphorylated by MAPKs, which serve as the central regulatory hubs in diverse plant signalling networks (Popescu *et al.*, 2009). A strikingly large number of putative MAPK cascade-encoding genes have been predicted in Arabidopsis (Ichimura *et al.*, 2002). Numerous intrinsic and extracellular stimuli can activate MAPK cascades to regulate gene expression and integrate developmental processes (Xu and Zhang, 2015; Komis *et al.*, 2018). However, current knowledge on the physiological functions of MAPK cascades in nutrient signalling is relatively limited (Chardin *et al.*, 2017).

The key TF NLP7 binds directly to the promoters of *MAPKKK13*, *14*, and *19*, which are the top nitrate-activated genes in many transcriptome datasets (Table 1). It will be interesting to define their downstream MAPKKs and MAPKs, which may promote nitrate signalling. Nitrate also activates other genes encoding putative RAF-like MAPKKKs, including INTEGRIN-LINKED KINASE 4 (ILK4), which interacts with RLKs and a putative calcium channel (Popescu *et al.*, 2017), as well as STY8 and STY46, which phosphorylate nuclear-encoded proteins targeted to the chloroplast (Lamberti *et al.*, 2011). STY8/46 may be important for nitrate-induced leaf greening and chloroplast development because mutations of *STY8, STY46*, and another homologous gene, *STY17*, in the *sty8,17,46* triple mutant result in reduced growth and chlorophyll accumulation. As nitrate consistently activates *STY46* but represses *STY8* (Table 1) and the *sty46* mutant displays severe growth retardation, *STY46* likely plays the predominant role in chloroplast and leaf development promoted by nitrate. The ambiguous effect of nitrate on the expression of *STY8/46* suggests that other stimuli or tissue-specific expression may contribute to the regulation of *STY8/46*.

Intriguingly, the expression of *MKK9* is repressed by nitrate in five experimental conditions (Table 1) and is induced by relatively low (0.1 mM) nitrate concentrations in 2–3 days, which probably corresponds to nitrate starvation conditions (Luo *et al.*, 2017). The expression of constitutively active MKK9 kinase in low-nitrate concentrations reduces the expression of anthocyanin biosynthesis genes as well as the accumulation of anthocyanin (Luo *et al.*, 2017). It was suggested that *MKK9* negatively regulates the production of anthocyanins but promotes the acquisition of nitrogen through the activation of *NRT2.1*. This finding is inconsistent with the role of MKK9 in promoting MPK6 activation in ethylene signalling and plant senescence promoted by nitrate starvation (Yoo *et al.*, 2009; Zhou *et al.*, 2009). A recent study indicated that MPK3 and MPK6 are required for controlling the proper progression of lateral root primordia (Zhu *et al.*, 2019). Whether nitrate modulates lateral root formation through MKK9–MPK3/6 signalling is unclear and requires further study. Finally, *MPK18* is activated by nitrate (Table 1), and phosphorylated MPK18 can interact with and be dephosphorylated by the dual-specific MAPK phosphatase PROPYZAMIDE HYPERSENSITIVE 1 (PHS1), which is involved in the stabilization of cortical microtubules (Walia *et al.*, 2009). As *mpk18* exhibits enhanced microtubule stability, future experiments may determine whether the MPK18–PHS1 signalling module governs the morphological response of the root system to nitrate (Fig. 2).

### Connections to RLKs

Although nitrate regulates a large number of genes encoding different subfamilies of RLKs, their responses to nitrate are highly variable in different transcriptome datasets (Table 1), which suggests that they have specialized roles in nitrate signalling in different cell types, organs, developmental stages, and growth conditions. More than 200 genes in the Arabidopsis genome encode homologs of leucine-rich repeat RLKs (LRR-RKs), the largest family of putative receptor kinases (Shiu and Bleecker, 2001; Chakraborty *et al.*, 2019). The extracellular LRR domains of some LRR-RKs mediate responses to endogenous peptides that modulate cell proliferation, cell differentiation, immunity, symbiosis, and the response to wounding. For instance, the perception of PLANT PEPTIDE CONTAINING SULFATED TYROSINE (PSY1) by the PSY1 receptor (PSY1R) is reported to promote cell proliferation in roots and cell expansion in the elongation zone of roots (Amano *et al.*, 2007). The up-regulation of the *PSY1R* gene in multiple microarray datasets (Table 1) may suggest its positive role in enhancing the growth of roots supplied with nitrate. Moreover, many genes encoding cysteine-rich RLKs (CRKs) are also induced by nitrate (Chen, 2001). *CRK29* and *CRK30* stand out as the most consistently up-regulated nitrate-responsive genes (Table 1). Although CRK29 and CRK30 were reported to be involved in plant immunity and cell-to-cell communication, the possible functions of CRK29/30 in nitrate signalling could be further explored (Amari *et al.*, 2010; Lee *et al.*, 2011; Caillaud *et al.*, 2014).

### Multiple roles of PP2Cs

Protein dephosphorylation is a crucial process for reversing protein phosphorylation and controlling intracellular signalling events. In Arabidopsis, around 112 PP genes have been predicted. Among these, there are 76 *PP2C* gene members that can be clustered into 10 groups (A to J) with the exception of six unclustered genes (Schweighofer *et al.*, 2004). There are 12 nitrate-induced *PP2C* genes (Table 1). *PP2CA* activation was detected in five microarray datasets (Table 1) and has been shown to negatively regulate ABA-mediated responses in seeds and at the vegetative stage, the kinase activity of the SNF1-RELATED PROTEIN KINASE 1 (SnRK1) energy sensor, and the guard cell outward-rectifying potassium efflux channel (Rodrigues *et al.*, 2013; Lefoulon *et al.*, 2016). SnRK1 is the central metabolic regulator of energy homeostasis in plants. PP2CA dephosphorylates SnRK1 and inhibits energy signalling, modulating growth, stress tolerance, and senescence (Rodrigues *et al.*, 2013). Investigation of the PP2CA–SnRK1 connection in nitrate signalling may uncover new roles for the energy sensor complexes in the PNR (Fig. 2).

The genes encoding PP2C-type phosphatases AP2C1 and PP2C5 are also activated by nitrate (Table 1). AP2C1 and PP2C5 share overlapping functions and can directly interact with and dephosphorylate MPK3/4/6 predominantly in the nucleus (Schweighofer *et al.*, 2007; Brock *et al.*, 2010). The *ap2c1* mutant produces more jasmonate upon wounding and is more resistant to phytophagous mites, whereas plants with increased AP2C1 levels reduce ethylene production and are more susceptible to a necrotrophic fungal pathogen (Schweighofer *et al.*, 2007). Unexpectedly, the analyses of *ap2c1*, *pp2c5*, and *ap2c1 pp2c5* mutants suggest that they have positive roles in seed germination, stomatal closure, and gene regulation in ABA responses (Brock *et al.*, 2010). Besides interacting with MPK3/4/6, AP2C1 dephosphorylates CIPK9 to regulate root growth and seedling development under low-potassium conditions (Singh *et al.*, 2018). Nitrate may induce PP2C-type phosphatases to modulate these important regulators to control or fine-tune plant growth. Further studies may clarify the functions of AP2C1 and PP2C5 in nitrate signalling as well as the complex nutrient–hormone crosstalk mediating growth, stress, and immune responses (Fig. 2).

## Nitrate-dependent phosphorylation of plant proteins

Complementary approaches to identify the relevant substrates of diverse PKs or PPs that are regulated by nitrate at the transcriptional or post-transcriptional level provide further molecular and biochemical insight into the PNR. Advances in phosphoproteomics analyses have enabled site-specific quantification of *in vivo* phosphorylation of a broad range of proteins (Engelsberger and Schulze, 2012; Li *et al.*, 2015). Two studies have generated quantitative data focusing on protein phosphorylation dynamics and phosphopeptide identification in response to nitrate or ammonium supply (Engelsberger and Schulze, 2012) or deprivation (Menz *et al.*, 2016). Within 5 min of nitrate resupply, the high-affinity nitrate transporter NRT2;1, ammonium transporter AMT1;1, and nitrate reductase NIA2 are rapidly dephosphorylated, while diverse PKs show increased phosphorylation (Engelsberger and Schulze, 2012). These findings are consistent with NIA2 phosphorylation at Ser-534 and NRT2.1 phosphorylation at Ser-28 under nitrate-deprivation conditions (Engelsberger and Schulze, 2012; Menz *et al.*, 2016). The highly conserved Ser-543 in NIA2 has been reported to exert an inhibitory function when it is phosphorylated (Menz *et al.*, 2016). Future research will identify more nitrate-regulated phosphorylation sites and their cognate PKs or PPs that are relevant to the regulation of transporter, sensor, channel, enzyme, or TF activities and the control of nitrate-dependent plant development.

Nitrate resupply stimulates the rapid phosphorylation of many interesting PKs, including the LRR-RKs IMPAIRED OOMYCETE SUSCEPTIBILITY 1 (IOS1) (Hok *et al.*, 2014) and KINASE7 (KIN7) (Isner *et al.*, 2018), the membrane-associated PKs CAST AWAY (CST) (Burr *et al.*, 2011; Groner *et al.*, 2016), CIPK2 (Linn *et al.*, 2017), osmotic stress regulator SnRK2.4 (McLoughlin *et al.*, 2012), BR-SIGNALING KINASE 1 (BSK1) (Tang *et al.*, 2008; Sreeramulu *et al.*, 2013), and the kinesin-associated PK NEVER IN MITOSIS GENE A-RELATED KINASE 6 (NEK6) (Takatani *et al.*, 2017) (Fig. 2). IOS1 and KIN7 could be involved in nitrate–ABA signalling crosstalk (Ondzighi-Assoume *et al.*, 2016; Ristova *et al.*, 2016). The *ios1* mutant plant shows hypersensitivity to ABA-induced inhibition of seed germination and primary root elongation (Hok *et al.*, 2014), and KIN7 participates in ABA-induced stomatal closure by modulating tonoplast potassium channel activity (Isner *et al.*, 2018). CST activates the cell separation process that may be required for the promotion by nitrate of lateral root primordia emergence (Burr *et al.*, 2011; Groner *et al.*, 2016; Zhu *et al.*, 2019).

SnRK2.4 links the perception of salt stress to the modulation of root growth and development (McLoughlin *et al.*, 2012). However, it is unclear how nitrate triggers SnRK2.4 phosphorylation and regulates root growth. Brassinosteroid (BR) promotes plant growth through the BR receptor (BRI)–BKS1 kinase relay to regulate BR signalling, and BRI1 phosphorylates BSK1 at Ser-230 (Tang *et al.*, 2008). As nitrate also induces Ser-230 phosphorylation of BSK1, future studies will elucidate the molecular mechanism integrating nitrate and BR signalling in the control of plant growth. NEK6 is a mitotic kinase that destabilizes cortical microtubules and functions in promoting longitudinal cell elongation and suppressing radial ectopic growth (Takatani *et al.*, 2017). Although nitrate could trigger the phosphorylation of diverse PKs, the molecular and physiological functions underlying these phosphorylation events are largely unknown. Elucidating the precise roles and regulations of nitrate-activated phosphorylation of these PKs via calcium-dependent and calcium-independent mechanisms will significantly expand the current scope of nitrate signalling in plants (Fig. 2).

## Perspectives

Here we consider the new and essential roles of calcium signalling and dynamic protein phosphorylation via diverse mechanisms in the PNR. Although most nitrate responses have been characterized in root systems, recent findings have demonstrated in both roots and shoots that nitrate triggers unique calcium signatures and acts synergistically with calcium-activated, nuclear-localized CPK10/30/32 to phosphorylate NLP7. The activated NLP7 is retained in the nucleus and specifies a global reprogramming of nitrate-responsive genes (Marchive *et al.*, 2013; Liu *et al.*, 2017). Besides NLPs, other TFs, such as ARABIDOPSIS NITRATE REGULATED 1, LBD37/38/39, TGA1/4, TCP20, HRS1/HRS1 Homolog 1, Bric-a-Brac/Tramtrack/Broad gene family 1/2, bZIP1, SPL9, and NAM/ATAF/CUC4, have been shown to control transcriptional responses to nitrate (Vidal *et al.*, 2015; Wang *et al.*, 2018; Brooks *et al.*, 2019). Future investigations could examine whether the regulation of these TFs is also dependent on CPKs and define the precise and extensive connections between specific CPKs and TFs in nitrate signalling. It is possible that other NLPs and TFs may also participate in the nitrate–calcium–CPK–TF signalling network that integrates the transcriptome, nutrient transport, cellular metabolism, hormone signalling, and cell proliferation, which mediate the shoot–root coordination and developmental plasticity that shape plant growth and development. Further studies may determine novel target genes and *cis*-regulatory elements for NLP7 and other NLPs and TFs in response to nitrate–calcium–CPK signalling or other upstream regulators in native biological contexts and cell types (Walker *et al.*, 2017).

The nitrate transceptor NPF6.3 and PLC–IP_3_ have been suggested to play key roles in nitrate-triggered calcium signalling (Fig. 1) (Riveras *et al.*, 2015). However, it cannot be ruled out that other nitrate transporter-related and novel nitrate-binding proteins may serve as nitrate sensors in the PNR. The examination of specific subcellular sites for nitrate-stimulated calcium signals based on targeted GCaMP6s may facilitate screening for and identification of new signalling molecules and calcium channels that activate nitrate responses (Yuan *et al.*, 2014; Jiang *et al.*, 2019). Although the nitrate-responsive expression of most genes associated with metabolism and transport depends on CPK10/30/32, CIPK8/CIPK23, in complex with putative CBL1/CBL9 calcium sensors, can regulate the transport activity of NPF6.3 and signalling downstream of both NPF6.3 and calcium (Fig. 2) (Ho *et al.*, 2009; Hu *et al.*, 2009). Integrated molecular, cellular, biochemical, and genetic analyses may identify additional nitrate sensors, calcium channels, calcium sensors, PKs, and PPs contributing to the PNR (Léran *et al.*, 2015; Liu *et al.*, 2017).

Global changes in gene expression were first reported for the PNR nearly two decades ago, and nitrate-induced genes encoding TFs have been shown to play key roles in regulating nitrate signalling (Vidal *et al.*, 2015; Wang *et al.*, 2018; Brooks *et al.*, 2019). However, the roles of only a few nitrate-regulated genes encoding putative PKs or PPs have been defined. In future investigations, functional screens might focus on these PK and PP genes as candidates to discover their roles in nitrate signalling and establish new nitrate-based molecular wiring in the gene regulatory network. Technical advances in the preparation and enrichment of samples and quantification strategies for phosphoproteomics could improve the scope and resolution of early protein phosphorylation events in the PNR. The identification of new targets of nitrate-activated CPKs, CIPKs, or other PKs could reveal additional components or connections in the PNR.

Nitrogen is the fourth most abundant element in plant biomass after hydrogen, carbon, and oxygen, and nitrate, the preferred source of inorganic nitrogen for most plants, is acquired from soil. The Green Revolution, which profoundly increased agricultural productivity worldwide, depends on the application of nitrogenous fertilizers to soil to ensure that the photosynthetic potential of crops is not limited by nitrogen assimilation and nitrate signalling. However, the production and use of fertilizer requires substantial energy consumption and represents a large economic burden. Moreover, the leaching of nitrate from the use of fertilizer pollutes waterways and promotes eutrophication. A better understanding of nitrate signalling and downstream responses could suggest rational ways to improve nitrogen utilization and sustain agriculture with reduced energy input and pollution.

## References

[CIT0001] Alvarez JM, Moyano TC, Zhang T, et al. 2019 Local changes in chromatin accessibility and transcriptional networks underlying the nitrate response in *Arabidopsis* roots. Molecular Plant 12, 1545–1560. 31526863 10.1016/j.molp.2019.09.002

[CIT0002] Amano Y, Tsubouchi H, Shinohara H, Ogawa M, Matsubayashi Y 2007 Tyrosine-sulfated glycopeptide involved in cellular proliferation and expansion in *Arabidopsis*. Proceedings of the National Academy of Sciences, USA 104, 18333–18338. 10.1073/pnas.0706403104 PMC2084343 17989228

[CIT0003] Amari K, Boutant E, Hofmann C, et al. 2010 A family of plasmodesmal proteins with receptor-like properties for plant viral movement proteins. PLoS Pathogens 6, e1001119. 20886105 10.1371/journal.ppat.1001119 PMC2944810

[CIT0004] Bang W, Kim S, Ueda A, Vikram M, Yun D, Bressan RA, Hasegawa PM, Bahk J, Koiwa H 2006 Arabidopsis carboxyl-terminal domain phosphatase-like isoforms share common catalytic and interaction domains but have distinct in planta functions. Plant Physiology 142, 586–594. 16905668 10.1104/pp.106.084939 PMC1586060

[CIT0005] Bender KW, Zielinski RE, Huber SC 2018 Revisiting paradigms of Ca^2+^ signaling protein kinase regulation in plants. Biochemical Journal 475, 207–223. 29305430 10.1042/BCJ20170022

[CIT0006] Berdy SE, Kudla J, Gruissem W, Gillaspy GE 2001 Molecular characterization of At5PTase1, an inositol phosphatase capable of terminating inositol trisphosphate signaling. Plant Physiology 126, 801–810. 11402208 10.1104/pp.126.2.801 PMC111170

[CIT0007] Berridge MJ 2016 The inositol trisphosphate/calcium signaling pathway in health and disease. Physiological Reviews 96, 1261–1296. 27512009 10.1152/physrev.00006.2016

[CIT0008] Bloom AJ 2015 Photorespiration and nitrate assimilation: a major intersection between plant carbon and nitrogen. Photosynthesis Research 123, 117–128. 25366830 10.1007/s11120-014-0056-y

[CIT0009] Boudsocq M, Droillard MJ, Regad L, Laurière C 2012 Characterization of *Arabidopsis* calcium-dependent protein kinases: activated or not by calcium? Biochemical Journal 447, 291–299. 22827269 10.1042/BJ20112072

[CIT0010] Boudsocq M, Sheen J 2013 CDPKs in immune and stress signaling. Trends in Plant Science 18, 30–40. 22974587 10.1016/j.tplants.2012.08.008 PMC3534830

[CIT0011] Brock AK, Willmann R, Kolb D, Grefen L, Lajunen HM, Bethke G, Lee J, Nürnberger T, Gust AA 2010 The Arabidopsis mitogen-activated protein kinase phosphatase PP2C5 affects seed germination, stomatal aperture, and abscisic acid-inducible gene expression. Plant Physiology 153, 1098–1111. 20488890 10.1104/pp.110.156109 PMC2899920

[CIT0012] Brooks MD, Cirrone J, Pasquino AV, et al. 2019 Network Walking charts transcriptional dynamics of nitrogen signaling by integrating validated and predicted genome-wide interactions. Nature Communications 10, 1569. 10.1038/s41467-019-09522-1 PMC6451032 30952851

[CIT0013] Buratowski S 2009 Progression through the RNA polymerase II CTD cycle. Molecular Cell 36, 541–546. 19941815 10.1016/j.molcel.2009.10.019 PMC3232742

[CIT0014] Burr CA, Leslie ME, Orlowski SK, Chen I, Wright CE, Daniels MJ, Liljegren SJ 2011 CAST AWAY, a membrane-associated receptor-like kinase, inhibits organ abscission in Arabidopsis. Plant Physiology 156, 1837–1850. 21628627 10.1104/pp.111.175224 PMC3149937

[CIT0015] Caillaud MC, Wirthmueller L, Sklenar J, Findlay K, Piquerez SJ, Jones AM, Robatzek S, Jones JD, Faulkner C 2014 The plasmodesmal protein PDLP1 localises to haustoria-associated membranes during downy mildew infection and regulates callose deposition. PLoS Pathogens 10, e1004496. 25393742 10.1371/journal.ppat.1004496 PMC4231120

[CIT0016] Canales J, Moyano TC, Villarroel E, Gutiérrez RA 2014 Systems analysis of transcriptome data provides new hypotheses about *Arabidopsis* root response to nitrate treatments. Frontiers in Plant Science 5, 22. 24570678 10.3389/fpls.2014.00022 PMC3917222

[CIT0017] Chakraborty S, Nguyen B, Wasti SD, Xu G 2019 Plant leucine-rich repeat receptor kinase (LRR-RK): structure, ligand perception, and activation mechanism. Molecules 24, E3081. 31450667 10.3390/molecules24173081 PMC6749341

[CIT0018] Chardin C, Schenk ST, Hirt H, Colcombet J, Krapp A 2017 Review: Mitogen-Activated Protein Kinases in nutritional signaling in *Arabidopsis*. Plant Science 260, 101–108. 28554467 10.1016/j.plantsci.2017.04.006

[CIT0019] Chen TW, Wardill TJ, Sun Y, et al. 2013 Ultrasensitive fluorescent proteins for imaging neuronal activity. Nature 499, 295–300. 23868258 10.1038/nature12354 PMC3777791

[CIT0020] Chen Z 2001 A superfamily of proteins with novel cysteine-rich repeats. Plant Physiology 126, 473–476. 11402176 10.1104/pp.126.2.473 PMC1540112

[CIT0021] Costa A, Navazio L, Szabo I 2018 The contribution of organelles to plant intracellular calcium signalling. Journal of Experimental Botany 69, 4175–4193. 10.1093/jxb/ery185 29767757

[CIT0022] Crawford NM, Forde BG 2002 Molecular and developmental biology of inorganic nitrogen nutrition. The Arabidopsis Book 1, e0011. 22303192 10.1199/tab.0011 PMC3243300

[CIT0023] Curran A, Chang IF, Chang CL, et al. 2011 Calcium-dependent protein kinases from *Arabidopsis* show substrate specificity differences in an analysis of 103 substrates. Frontiers in Plant Science 2, 36. 22645532 10.3389/fpls.2011.00036 PMC3355778

[CIT0024] Dai N, Wang W, Patterson SE, Bleecker AB 2013 The TMK subfamily of receptor-like kinases in *Arabidopsis* display an essential role in growth and a reduced sensitivity to auxin. PLoS One 8, e60990. 23613767 10.1371/journal.pone.0060990 PMC3628703

[CIT0025] Dana H, Sun Y, Mohar B, et al. 2019 High-performance calcium sensors for imaging activity in neuronal populations and microcompartments. Nature Methods 16, 649–657. 31209382 10.1038/s41592-019-0435-6

[CIT0026] Dechorgnat J, Nguyen CT, Armengaud P, Jossier M, Diatloff E, Filleur S, Daniel-Vedele F 2011 From the soil to the seeds: the long journey of nitrate in plants. Journal of Experimental Botany 62, 1349–1359. 21193579 10.1093/jxb/erq409

[CIT0027] Dodd AN, Kudla J, Sanders D 2010 The language of calcium signaling. Annual Review of Plant Biology 61, 593–620. 10.1146/annurev-arplant-070109-104628 20192754

[CIT0028] Engelsberger WR, Schulze WX 2012 Nitrate and ammonium lead to distinct global dynamic phosphorylation patterns when resupplied to nitrogen-starved Arabidopsis seedlings. The Plant Journal 69, 978–995. 22060019 10.1111/j.1365-313X.2011.04848.x PMC3380553

[CIT0029] Fredes I, Moreno S, Díaz FP, Gutiérrez RA 2019 Nitrate signaling and the control of Arabidopsis growth and development. Current Opinion in Plant Biology 47, 112–118. 30496968 10.1016/j.pbi.2018.10.004

[CIT0030] Gaudinier A, Rodriguez-Medina J, Zhang L, et al. 2018 Transcriptional regulation of nitrogen-associated metabolism and growth. Nature 563, 259–264. 30356219 10.1038/s41586-018-0656-3

[CIT0031] Gifford ML, Dean A, Gutierrez RA, Coruzzi GM, Birnbaum KD 2008 Cell-specific nitrogen responses mediate developmental plasticity. Proceedings of the National Academy of Sciences, USA 105, 803–808. 10.1073/pnas.0709559105 PMC2206617 18180456

[CIT0032] Grant M, Brown I, Adams S, Knight M, Ainslie A, Mansfield J 2000 The *RPM1* plant disease resistance gene facilitates a rapid and sustained increase in cytosolic calcium that is necessary for the oxidative burst and hypersensitive cell death. The Plant Journal 23, 441–450. 10972870 10.1046/j.1365-313x.2000.00804.x

[CIT0033] Groner WD, Christy ME, Kreiner CM, Liljegren SJ 2016 Allele-specific interactions between *CAST AWAY* and *NEVERSHED* control abscission in *Arabidopsis* flowers. Frontiers in Plant Science 7, 1588. 27818674 10.3389/fpls.2016.01588 PMC5073242

[CIT0034] Gunesekera B, Torabinejad J, Robinson J, Gillaspy GE 2007 Inositol polyphosphate 5-phosphatases 1 and 2 are required for regulating seedling growth. Plant Physiology 143, 1408–1417. 17237190 10.1104/pp.106.089474 PMC1820906

[CIT0035] Guo FQ, Young J, Crawford NM 2003 The nitrate transporter AtNRT1.1 (CHL1) functions in stomatal opening and contributes to drought susceptibility in Arabidopsis. The Plant Cell 15, 107–117. 12509525 10.1105/tpc.006312 PMC143464

[CIT0036] Gutiérrez RA, Lejay LV, Dean A, Chiaromonte F, Shasha DE, Coruzzi GM 2007 Qualitative network models and genome-wide expression data define carbon/nitrogen-responsive molecular machines in *Arabidopsis*. Genome Biology 8, R7. 17217541 10.1186/gb-2007-8-1-r7 PMC1839130

[CIT0037] Ho CH, Lin SH, Hu HC, Tsay YF 2009 CHL1 functions as a nitrate sensor in plants. Cell 138, 1184–1194. 19766570 10.1016/j.cell.2009.07.004

[CIT0038] Hok S, Allasia V, Andrio E, et al. 2014 The receptor kinase IMPAIRED OOMYCETE SUSCEPTIBILITY1 attenuates abscisic acid responses in Arabidopsis. Plant Physiology 166, 1506–1518. 25274985 10.1104/pp.114.248518 PMC4226379

[CIT0039] Hu B, Jiang Z, Wang W, et al. 2019 Nitrate–NRT1.1B–SPX4 cascade integrates nitrogen and phosphorus signalling networks in plants. Nature Plants 5, 401–413. 30911122 10.1038/s41477-019-0384-1

[CIT0040] Hu HC, Wang YY, Tsay YF 2009 AtCIPK8, a CBL-interacting protein kinase, regulates the low-affinity phase of the primary nitrate response. The Plant Journal 57, 264–278. 18798873 10.1111/j.1365-313X.2008.03685.x

[CIT0041] Ichimura K, Shinozaki K, Tena G, et al 2002 Mitogen-activated protein kinase cascades in plants: a new nomenclature. Trends in Plant Science 7, 301–308. 12119167 10.1016/s1360-1385(02)02302-6

[CIT0042] Isner JC, Begum A, Nuehse T, Hetherington AM, Maathuis FJM 2018 KIN7 kinase regulates the vacuolar TPK1 K^+^ channel during stomatal closure. Current Biology 28, 466–472. 29395926 10.1016/j.cub.2017.12.046

[CIT0043] Jiang Z, Zhou X, Tao M, et al. 2019 Plant cell-surface GIPC sphingolipids sense salt to trigger Ca^2+^ influx. Nature 572, 341–346. 31367039 10.1038/s41586-019-1449-z

[CIT0044] Kanehara K, Yu CY, Cho Y, Cheong WF, Torta F, Shui G, Wenk MR, Nakamura Y 2015 Arabidopsis AtPLC2 is a primary phosphoinositide-specific phospholipase C in phosphoinositide metabolism and the endoplasmic reticulum stress response. PLoS Genetics 11, e1005511. 26401841 10.1371/journal.pgen.1005511 PMC4581737

[CIT0045] Kiba T, Krapp A 2016 Plant nitrogen acquisition under low availability: regulation of uptake and root architecture. Plant & Cell Physiology 57, 707–714. 27025887 10.1093/pcp/pcw052 PMC4836452

[CIT0046] Kiba T, Takei K, Kojima M, Sakakibara H 2013 Side-chain modification of cytokinins controls shoot growth in *Arabidopsis*. Developmental Cell 27, 452–461. 24286826 10.1016/j.devcel.2013.10.004

[CIT0047] Knight H, Trewavas AJ, Knight MR 1996 Cold calcium signaling in Arabidopsis involves two cellular pools and a change in calcium signature after acclimation. The Plant Cell 8, 489–503. 8721751 10.1105/tpc.8.3.489 PMC161115

[CIT0048] Kolukisaoglu U, Weinl S, Blazevic D, Batistic O, Kudla J 2004 Calcium sensors and their interacting protein kinases: genomics of the Arabidopsis and rice CBL-CIPK signaling networks. Plant Physiology 134, 43–58. 14730064 10.1104/pp.103.033068 PMC316286

[CIT0049] Komis G, Šamajová O, Ovečka M, Šamaj J 2018 Cell and developmental biology of plant mitogen-activated protein kinases. Annual Review of Plant Biology 69, 237–265. 10.1146/annurev-arplant-042817-040314 29489398

[CIT0050] Konishi M, Yanagisawa S 2013 Arabidopsis NIN-like transcription factors have a central role in nitrate signalling. Nature Communications 4, 1617. 10.1038/ncomms2621 23511481

[CIT0051] Krouk G, Lacombe B, Bielach A, et al. 2010a Nitrate-regulated auxin transport by NRT1.1 defines a mechanism for nutrient sensing in plants. Developmental Cell 18, 927–937. 20627075 10.1016/j.devcel.2010.05.008

[CIT0052] Krouk G, Mirowski P, LeCun Y, Shasha DE, Coruzzi GM 2010b Predictive network modeling of the high-resolution dynamic plant transcriptome in response to nitrate. Genome Biology 11, R123. 21182762 10.1186/gb-2010-11-12-r123 PMC3046483

[CIT0053] Lamberti G, Gügel IL, Meurer J, Soll J, Schwenkert S 2011 The cytosolic kinases STY8, STY17, and STY46 are involved in chloroplast differentiation in Arabidopsis. Plant Physiology 157, 70–85. 21799034 10.1104/pp.111.182774 PMC3165899

[CIT0054] Lee JY, Wang X, Cui W, et al. 2011 A plasmodesmata-localized protein mediates crosstalk between cell-to-cell communication and innate immunity in *Arabidopsis*. The Plant Cell 23, 3353–3373. 21934146 10.1105/tpc.111.087742 PMC3203451

[CIT0055] Lefoulon C, Boeglin M, Moreau B, Véry AA, Szponarski W, Dauzat M, Michard E, Gaillard I, Chérel I 2016 The *Arabidopsis* AtPP2CA protein phosphatase inhibits the GORK K^+^ efflux channel and exerts a dominant suppressive effect on phosphomimetic-activating mutations. Journal of Biological Chemistry 291, 6521–6533. 26801610 10.1074/jbc.M115.711309 PMC4813591

[CIT0056] Léran S, Edel KH, Pervent M, Hashimoto K, Corratgé-Faillie C, Offenborn JN, Tillard P, Gojon A, Kudla J, Lacombe B 2015 Nitrate sensing and uptake in *Arabidopsis* are enhanced by ABI2, a phosphatase inactivated by the stress hormone abscisic acid. Science Signaling 8, ra43. 25943353 10.1126/scisignal.aaa4829

[CIT0057] Li F, Cheng C, Cui F, et al. 2014 Modulation of RNA polymerase II phosphorylation downstream of pathogen perception orchestrates plant immunity. Cell Host & Microbe 16, 748–758. 25464831 10.1016/j.chom.2014.10.018 PMC4268009

[CIT0058] Li J, Silva-Sanchez C, Zhang T, Chen S, Li H 2015 Phosphoproteomics technologies and applications in plant biology research. Frontiers in Plant Science 6, 430. 26136758 10.3389/fpls.2015.00430 PMC4468387

[CIT0059] Li Z, Wang R, Gao Y, et al. 2017 The *Arabidopsis CPSF30-L* gene plays an essential role in nitrate signaling and regulates the nitrate transceptor gene *NRT1.1*. New Phytologist 216, 1205–1222. 28850721 10.1111/nph.14743

[CIT0060] Lin YL, Tsay YF 2017 Influence of differing nitrate and nitrogen availability on flowering control in Arabidopsis. Journal of Experimental Botany 68, 2603–2609. 28369493 10.1093/jxb/erx053

[CIT0061] Linkohr BI, Williamson LC, Fitter AH, Leyser HM 2002 Nitrate and phosphate availability and distribution have different effects on root system architecture of *Arabidopsis*. The Plant Journal 29, 751–760. 12148533 10.1046/j.1365-313x.2002.01251.x

[CIT0062] Linn J, Ren M, Berkowitz O, Ding W, van der Merwe MJ, Whelan J, Jost R 2017 Root cell-specific regulators of phosphate-dependent growth. Plant Physiology 174, 1969–1989. 28465462 10.1104/pp.16.01698 PMC5490885

[CIT0063] Liu KH, McCormack M, Sheen J 2012 Targeted parallel sequencing of large genetically-defined genomic regions for identifying mutations in *Arabidopsis*. Plant Methods 8, 12. 22462410 10.1186/1746-4811-8-12 PMC3348062

[CIT0064] Liu KH, Niu Y, Konishi M, et al. 2017 Discovery of nitrate–CPK–NLP signalling in central nutrient–growth networks. Nature 545, 311–316. 28489820 10.1038/nature22077 PMC5823009

[CIT0065] Liu KH, Tsay YF 2003 Switching between the two action modes of the dual-affinity nitrate transporter CHL1 by phosphorylation. The EMBO Journal 22, 1005–1013. 12606566 10.1093/emboj/cdg118 PMC150351

[CIT0066] Luan S 2009 The CBL–CIPK network in plant calcium signaling. Trends in Plant Science 14, 37–42. 19054707 10.1016/j.tplants.2008.10.005

[CIT0067] Luo J, Wang X, Feng L, Li Y, He JX 2017 The mitogen-activated protein kinase kinase 9 (MKK9) modulates nitrogen acquisition and anthocyanin accumulation under nitrogen-limiting condition in *Arabidopsis*. Biochemical and Biophysical Research Communications 487, 539–544. 28435067 10.1016/j.bbrc.2017.04.065

[CIT0068] Ma W, Li J, Qu B, He X, Zhao X, Li B, Fu X, Tong Y 2014 Auxin biosynthetic gene *TAR2* is involved in low nitrogen-mediated reprogramming of root architecture in Arabidopsis. The Plant Journal 78, 70–79. 24460551 10.1111/tpj.12448

[CIT0069] Mao J, Nuruzzaman Manik SM, Shi S, Chao J, Jin Y, Wang Q, Liu H 2016 Mechanisms and physiological roles of the CBL-CIPK networking system in *Arabidopsis thaliana*. Genes 7, 62. 10.3390/genes7090062 PMC5042392 27618104

[CIT0070] Marchive C, Roudier F, Castaings L, Bréhaut V, Blondet E, Colot V, Meyer C, Krapp A 2013 Nuclear retention of the transcription factor NLP7 orchestrates the early response to nitrate in plants. Nature Communications 4, 1713. 10.1038/ncomms2650 23591880

[CIT0071] McLoughlin F, Galvan-Ampudia CS, Julkowska MM, Caarls L, van der Does D, Laurière C, Munnik T, Haring MA, Testerink C 2012 The Snf1-related protein kinases SnRK2.4 and SnRK2.10 are involved in maintenance of root system architecture during salt stress. The Plant Journal 72, 436–449. 22738204 10.1111/j.1365-313X.2012.05089.x PMC3533798

[CIT0072] Medici A, Krouk G 2014 The primary nitrate response: a multifaceted signalling pathway. Journal of Experimental Botany 65, 5567–5576. 24942915 10.1093/jxb/eru245

[CIT0073] Menz J, Li Z, Schulze WX, Ludewig U 2016 Early nitrogen-deprivation responses in Arabidopsis roots reveal distinct differences on transcriptome and (phospho-) proteome levels between nitrate and ammonium nutrition. The Plant Journal 88, 717–734. 27419465 10.1111/tpj.13272

[CIT0074] O’Brien JA, Vega A, Bouguyon E, Krouk G, Gojon A, Coruzzi G, Gutiérrez RA 2016 Nitrate transport, sensing, and responses in plants. Molecular Plant 9, 837–856. 27212387 10.1016/j.molp.2016.05.004

[CIT0075] Ondzighi-Assoume CA, Chakraborty S, Harris JM 2016 Environmental nitrate stimulates abscisic acid accumulation in Arabidopsis root tips by releasing it from inactive stores. The Plant Cell 28, 729–745. 26887919 10.1105/tpc.15.00946 PMC4826012

[CIT0076] Pandey GK, Grant JJ, Cheong YH, Kim BG, Li le G, Luan S 2008 Calcineurin-B-like protein CBL9 interacts with target kinase CIPK3 in the regulation of ABA response in seed germination. Molecular Plant 1, 238–248. 19825536 10.1093/mp/ssn003

[CIT0077] Patterson K, Cakmak T, Cooper A, Lager I, Rasmusson AG, Escobar MA 2010 Distinct signalling pathways and transcriptome response signatures differentiate ammonium- and nitrate-supplied plants. Plant, Cell & Environment 33, 1486–1501. 10.1111/j.1365-3040.2010.02158.x PMC2920365 20444219

[CIT0078] Perera IY, Hung CY, Brady S, Muday GK, Boss WF 2006 A universal role for inositol 1,4,5-trisphosphate-mediated signaling in plant gravitropism. Plant Physiology 140, 746–760. 16384898 10.1104/pp.105.075119 PMC1361340

[CIT0079] Popescu SC, Brauer EK, Dimlioglu G, Popescu GV 2017 Insights into the structure, function, and ion-mediated signaling pathways transduced by plant integrin-linked kinases. Frontiers in Plant Science 8, 376. 28421082 10.3389/fpls.2017.00376 PMC5376563

[CIT0080] Popescu SC, Popescu GV, Bachan S, Zhang Z, Gerstein M, Snyder M, Dinesh-Kumar SP 2009 MAPK target networks in *Arabidopsis thaliana* revealed using functional protein microarrays. Genes & Development 23, 80–92. 19095804 10.1101/gad.1740009 PMC2632172

[CIT0081] Poza-Carrión C, Paz-Ares J 2019 When nitrate and phosphate sensors meet. Nature Plants 5, 339–340. 30911124 10.1038/s41477-019-0403-2

[CIT0082] Ranf S, Gisch N, Schäffer M, et al. 2015 A lectin S-domain receptor kinase mediates lipopolysaccharide sensing in *Arabidopsis thaliana*. Nature Immunology 16, 426–433. 25729922 10.1038/ni.3124

[CIT0083] Ristova D, Carré C, Pervent M, et al. 2016 Combinatorial interaction network of transcriptomic and phenotypic responses to nitrogen and hormones in the *Arabidopsis thaliana* root. Science Signaling 9, rs13. 27811143 10.1126/scisignal.aaf2768

[CIT0084] Riveras E, Alvarez JM, Vidal EA, Oses C, Vega A, Gutiérrez RA 2015 The calcium ion is a second messenger in the nitrate signaling pathway of Arabidopsis. Plant Physiology 169, 1397–1404. 26304850 10.1104/pp.15.00961 PMC4587466

[CIT0085] Rodrigues A, Adamo M, Crozet P, et al. 2013 ABI1 and PP2CA phosphatases are negative regulators of Snf1-related protein kinase1 signaling in *Arabidopsis*. The Plant Cell 25, 3871–3884. 24179127 10.1105/tpc.113.114066 PMC3877788

[CIT0086] Ruffel S, Krouk G, Ristova D, Shasha D, Birnbaum KD, Coruzzi GM 2011 Nitrogen economics of root foraging: transitive closure of the nitrate–cytokinin relay and distinct systemic signaling for N supply vs. demand. Proceedings of the National Academy of Sciences, USA 108, 18524–18529. 10.1073/pnas.1108684108 PMC3215050 22025711

[CIT0087] Sakakibara H, Kobayashi K, Deji A, Sugiyama T 1997 Partial characterization of the signaling pathway for the nitrate-dependent expression of genes for nitrogen-assimilatory enzymes using detached maize leaves. Plant & Cell Physiology 38, 837–843.

[CIT0088] Sato T, Maekawa S, Yasuda S, et al. 2009 CNI1/ATL31, a RING-type ubiquitin ligase that functions in the carbon/nitrogen response for growth phase transition in Arabidopsis seedlings. The Plant Journal 60, 852–864. 19702666 10.1111/j.1365-313X.2009.04006.x

[CIT0089] Scheible WR, Morcuende R, Czechowski T, Fritz C, Osuna D, Palacios-Rojas N, Schindelasch D, Thimm O, Udvardi MK, Stitt M 2004 Genome-wide reprogramming of primary and secondary metabolism, protein synthesis, cellular growth processes, and the regulatory infrastructure of Arabidopsis in response to nitrogen. Plant Physiology 136, 2483–2499. 15375205 10.1104/pp.104.047019 PMC523316

[CIT0090] Schweighofer A, Hirt H, Meskiene I 2004 Plant PP2C phosphatases: emerging functions in stress signaling. Trends in Plant Science 9, 236–243. 15130549 10.1016/j.tplants.2004.03.007

[CIT0091] Schweighofer A, Kazanaviciute V, Scheikl E, et al. 2007 The PP2C-type phosphatase AP2C1, which negatively regulates MPK4 and MPK6, modulates innate immunity, jasmonic acid, and ethylene levels in *Arabidopsis*. The Plant Cell 19, 2213–2224. 17630279 10.1105/tpc.106.049585 PMC1955703

[CIT0092] Shiu SH, Bleecker AB 2001 Receptor-like kinases from *Arabidopsis* form a monophyletic gene family related to animal receptor kinases. Proceedings of the National Academy of Sciences, USA 98, 10763–10768. 10.1073/pnas.181141598 PMC58549 11526204

[CIT0093] Simeunovic A, Mair A, Wurzinger B, Teige M 2016 Know where your clients are: subcellular localization and targets of calcium-dependent protein kinases. Journal of Experimental Botany 67, 3855–3872. 27117335 10.1093/jxb/erw157

[CIT0094] Singh A, Bhatnagar N, Pandey A, Pandey GK 2015 Plant phospholipase C family: regulation and functional role in lipid signaling. Cell Calcium 58, 139–146. 25933832 10.1016/j.ceca.2015.04.003

[CIT0095] Singh A, Yadav AK, Kaur K, et al. 2018 A protein phosphatase 2C, AP2C1, interacts with and negatively regulates the function of CIPK9 under potassium-deficient conditions in Arabidopsis. Journal of Experimental Botany 69, 4003–4015. 29767755 10.1093/jxb/ery182 PMC6054203

[CIT0096] Sreeramulu S, Mostizky Y, Sunitha S, et al. 2013 BSKs are partially redundant positive regulators of brassinosteroid signaling in Arabidopsis. The Plant Journal 74, 905–919. 23496207 10.1111/tpj.12175

[CIT0097] Stitt M 1999 Nitrate regulation of metabolism and growth. Current Opinion in Plant Biology 2, 178–186. 10375569 10.1016/S1369-5266(99)80033-8

[CIT0098] Sueyoshi K, Mitsuyama T, Sugimoto T, Kleinhofs A, Warner R, Oji Y 1999 Effects of inhibitors for signaling components on the expression of the genes for nitrate reductase and nitrite reductase in excised barley leaves. Soil Science and Plant Nutrition 45, 1015–1019.

[CIT0099] Takatani S, Ozawa S, Yagi N, Hotta T, Hashimoto T, Takahashi Y, Takahashi T, Motose H 2017 Directional cell expansion requires NIMA-related kinase 6 (NEK6)-mediated cortical microtubule destabilization. Scientific Reports 7, 7826. 28798328 10.1038/s41598-017-08453-5 PMC5552743

[CIT0100] Tang W, Kim TW, Oses-Prieto JA, Sun Y, Deng Z, Zhu S, Wang R, Burlingame AL, Wang ZY 2008 BSKs mediate signal transduction from the receptor kinase BRI1 in *Arabidopsis*. Science 321, 557–560. 18653891 10.1126/science.1156973 PMC2730546

[CIT0101] Varala K, Marshall-Colón A, Cirrone J, et al. 2018 Temporal transcriptional logic of dynamic regulatory networks underlying nitrogen signaling and use in plants. Proceedings of the National Academy of Sciences, USA 115, 6494–6499. 10.1073/pnas.1721487115 PMC6016767 29769331

[CIT0102] Vidal EA, Álvarez JM, Moyano TC, Gutiérrez RA 2015 Transcriptional networks in the nitrate response of *Arabidopsis thaliana*. Current opinion in plant biology 27, 125–132. 26247122 10.1016/j.pbi.2015.06.010

[CIT0103] Vidal EA, Araus V, Lu C, Parry G, Green PJ, Coruzzi GM, Gutiérrez RA 2010 Nitrate-responsive miR393/*AFB3* regulatory module controls root system architecture in *Arabidopsis thaliana*. Proceedings of the National Academy of Sciences, USA 107, 4477–4482. 10.1073/pnas.0909571107 PMC2840086 20142497

[CIT0104] Walia A, Lee JS, Wasteneys G, Ellis B 2009 Arabidopsis mitogen-activated protein kinase MPK18 mediates cortical microtubule functions in plant cells. The Plant Journal 59, 565–575. 19392697 10.1111/j.1365-313X.2009.03895.x

[CIT0105] Walker L, Boddington C, Jenkins D, et al. 2017 Changes in gene expression in space and time orchestrate environmentally mediated shaping of root architecture. The Plant Cell 29, 2393–2412. 28893852 10.1105/tpc.16.00961 PMC5774560

[CIT0106] Wang R, Guegler K, LaBrie ST, Crawford NM 2000 Genomic analysis of a nutrient response in Arabidopsis reveals diverse expression patterns and novel metabolic and potential regulatory genes induced by nitrate. The Plant Cell 12, 1491–1509. 10948265 10.1105/tpc.12.8.1491 PMC149118

[CIT0107] Wang R, Okamoto M, Xing X, Crawford NM 2003 Microarray analysis of the nitrate response in Arabidopsis roots and shoots reveals over 1,000 rapidly responding genes and new linkages to glucose, trehalose-6-phosphate, iron, and sulfate metabolism. Plant Physiology 132, 556–567. 12805587 10.1104/pp.103.021253 PMC166997

[CIT0108] Wang R, Tischner R, Gutiérrez RA, Hoffman M, Xing X, Chen M, Coruzzi G, Crawford NM 2004 Genomic analysis of the nitrate response using a nitrate reductase-null mutant of Arabidopsis. Plant Physiology 136, 2512–2522. 15333754 10.1104/pp.104.044610 PMC523318

[CIT0109] Wang R, Xing X, Crawford N 2007 Nitrite acts as a transcriptome signal at micromolar concentrations in Arabidopsis roots. Plant Physiology 145, 1735–1745. 17951451 10.1104/pp.107.108944 PMC2151675

[CIT0110] Wang YY, Cheng YH, Chen KE, Tsay YF 2018 Nitrate transport, signaling, and use efficiency. Annual Review of Plant Biology 69, 85–122. 10.1146/annurev-arplant-042817-040056 29570365

[CIT0111] Widiez T, El Kafafi el S, Girin T, et al. 2011 HIGH NITROGEN INSENSITIVE 9 (HNI9)-mediated systemic repression of root NO_3_^–^ uptake is associated with changes in histone methylation. Proceedings of the National Academy of Sciences, USA 108, 13329–13334. 10.1073/pnas.1017863108 PMC3156160 21788519

[CIT0112] Xu G, Fan X, Miller AJ 2012 Plant nitrogen assimilation and use efficiency. Annual Review of Plant Biology 63, 153–182. 10.1146/annurev-arplant-042811-105532 22224450

[CIT0113] Xu J, Li HD, Chen LQ, Wang Y, Liu LL, He L, Wu WH 2006 A protein kinase, interacting with two calcineurin B-like proteins, regulates K^+^ transporter AKT1 in *Arabidopsis*. Cell 125, 1347–1360. 16814720 10.1016/j.cell.2006.06.011

[CIT0114] Xu J, Zhang S 2015 Mitogen-activated protein kinase cascades in signaling plant growth and development. Trends in Plant Science 20, 56–64. 25457109 10.1016/j.tplants.2014.10.001

[CIT0115] Yan D, Easwaran V, Chau V, et al. 2016 NIN-like protein 8 is a master regulator of nitrate-promoted seed germination in *Arabidopsis*. Nature Communications 7, 13179. 10.1038/ncomms13179 PMC5064020 27731416

[CIT0116] Yip Delormel T, Boudsocq M 2019 Properties and functions of calcium-dependent protein kinases and their relatives in *Arabidopsis thaliana*. New Phytologist 224, 585–604. 31369160 10.1111/nph.16088

[CIT0117] Yoo SD, Cho Y, Sheen J 2009 Emerging connections in the ethylene signaling network. Trends in Plant Science 14, 270–279. 19375376 10.1016/j.tplants.2009.02.007 PMC3063992

[CIT0118] Yuan F, Yang H, Xue Y, et al. 2014 OSCA1 mediates osmotic-stress-evoked Ca^2+^ increases vital for osmosensing in *Arabidopsis*. Nature 514, 367–371. 25162526 10.1038/nature13593

[CIT0119] Zhang H, Forde BG 2000 Regulation of *Arabidopsis* root development by nitrate availability. Journal of Experimental Botany 51, 51–59. 10938795

[CIT0120] Zhou C, Cai Z, Guo Y, Gan S 2009 An Arabidopsis mitogen-activated protein kinase cascade, MKK9-MPK6, plays a role in leaf senescence. Plant Physiology 150, 167–177. 19251906 10.1104/pp.108.133439 PMC2675715

[CIT0121] Zhu JK 2003 Regulation of ion homeostasis under salt stress. Current Opinion in Plant Biology 6, 441–445. 12972044 10.1016/s1369-5266(03)00085-2

[CIT0122] Zhu Q, Shao Y, Ge S, Zhang M, Zhang T, Hu X, Liu Y, Walker J, Zhang S, Xu J 2019 A MAPK cascade downstream of IDA–HAE/HSL2 ligand–receptor pair in lateral root emergence. Nature Plants 5, 414–423. 30936437 10.1038/s41477-019-0396-x

